# Outcomes of the Anterior-Based Muscle-Sparing Approach in Elective Total Hip Arthroplasty in Nonagenarians

**DOI:** 10.1016/j.artd.2023.101125

**Published:** 2023-05-11

**Authors:** Thomas M. Zink, George M. Babikian, Johanna M. Mackenzie, Callahan Sturgeon, Adam J. Rana, Brian J. McGrory

**Affiliations:** a Tufts University School of Medicine, Boston, MA, USA; b Tufts Medical Center, Boston, MA, USA; c Maine Medical Center, Portland, ME, USA

**Keywords:** Total hip arthroplasty (THA), Anterior-based muscle-sparing (ABMS) approach, Nonagenarian, Outcomes, ABLE

## Abstract

**Background:**

As the population ages, total hip arthroplasty has become more common in elderly patients including patients over the age of 90 years. Efficacy in this age group has been established, though literature regarding safety of total hip arthroplasty in nonagenarians is mixed. The anterior-based muscle-sparing (ABMS) approach, which exploits the intermuscular plane between the tensor fasciae latae and the gluteus medius, has proposed benefits of fast recovery, excellent stability, and reduced bleeding and may be adventitious among elderly, more fragile patients.

**Methods:**

A total of 38 consecutive nonagenarians undergoing elective, primary total hip arthroplasty via the ABMS approach for any indication from 2013 to 2020 were identified, and information regarding operative outcomes and patient-reported outcomes was gathered from review of medical records and our institutional joint replacement outcomes database.

**Results:**

Included patients ranged from 90 to 97 years of age with the majority classified as American Society of Anesthesiologists score 2 (50%) or American Society of Anesthesiologists 3 (47.4%). The mean operative time was 74.6 minutes ± 13.6 minutes. Of all patients, 5 required a transfusion, 2 patients were readmitted within 90 days, and there were no major complications. The mean hospital length of stay was 2.8 days ± 0.8 days with 22 patients (57.9%) discharged to a skilled nursing facility. Limited patient-reported outcomes data showed statistically significant improvements in most outcomes scores at 6 months to 1 year postoperatively compared to preoperative scores.

**Conclusions:**

The ABMS approach is safe and effective in nonagenarians who may benefit from decreased amounts of bleeding and recovery times associated with the ABMS approach, which is evident from the low complication rates, relatively short hospital lengths of stay, and acceptable transfusion rates compared to previous studies.

## Introduction

Total hip arthroplasty (THA) is a common and successful procedure, which offers the possibility to greatly improve mobility and quality of life. As the population ages, THA has been increasingly performed in older patients. Nonagenarians, people aged 90 years and older, are of particular interest as they comprise some of the oldest patients, and their numbers are expected to double to nearly 10% of the population by 2050 [1]. With this increase, the demand for THA in this age group is also expected to grow. While the efficacy of THA in nonagenarians is well established [[2], [3], [4], [5], [6]], literature regarding safety in this age group is mixed [[7], [8], [9], [10], [11], [12], [13], [14]] and there is a paucity of evidence regarding operative factors such as implant choice or surgical approach in this age group.

Historically, the posterior (Southern) approach has been a popular choice for THA due to its expansile nature, although the modified Hardinge approach has been frequently utilized due to a low incidence of dislocation. The direct anterior (DA) approach is gaining popularity with patients and surgeons with proposed benefits faster recovery and lower early dislocation risk. Another notable approach is the anterior-based muscle-sparing (ABMS) approach, ABLE, or Rottinger approach. Repopularized by Bertin and Rottinger in 2004, the ABMS approach to the hip is an intermuscular approach using the interval between the tensor fascia latae and gluteus medius [15,16]. While some studies have highlighted the learning curve for this surgery, there is evidence of decreased blood loss and quicker recovery of muscle strength, and among surgeons familiar with the approach, the operative times and complications appear to be similar or improved compared to other approaches [[17], [18], [19]]. To our knowledge, there are currently no published data regarding outcomes of the ABMS approach specifically among nonagenarians, with very limited data regarding specific approaches in nonagenarians in general. We therefore present a retrospective case series examining the outcomes of nonagenarians undergoing THA using the ABMS approach and seek to compare these data with currently published outcomes of THA in patients in their 10th decade of life.

## Material and methods

The study protocol was deemed exempt by our institutional review board who determined the project was exempt from institutional review board review. The electronic medical record was queried for patients aged 90 years and older at time of surgery undergoing elective THA using the ABMS approach for any indication from 2013 to 2020. Surgeries were performed by 1 of 3 arthroplasty surgeons experienced in the ABMS approach with use of cemented or press-fit implants performed at the surgeon’s discretion. The electronic medical record medical and functional outcomes measures, including Hip Dysfunction and Osteoarthritis Outcome Score for Joint Replacement (HOOS JR) scores, University of California, Los Angeles (UCLA) Activity Scores, Single Assessment Numerical Evaluation (SANE) scores, and Patient-Reported Outcomes Measurement Information System (PROMIS) physical and mental health scores, were extracted for the included patients from the center’s joint replacement outcomes database.

### Patient demographics

A total of 38 patients were included in the study, all undergoing elective unilateral THA (Table 1). The patients ranged from 90 to 97 years of age at the time of surgery with a mean age of 91.7 years. Patients tended to have multiple comorbidities with a median American Society of Anesthesiologists (ASA) score of 2.5 (Table 1). There were no patients with an ASA score of 1. The majority of patients had a body mass index (BMI) within the normal range with a mean BMI of 25.2. Among our set of 38 patients undergoing primary total hip arthroplasty, 36 patients (92.1%) underwent surgery for primary osteoarthritis and 2 patients (5.3%) for osteonecrosis of the femoral head. Of 38 patients, 23 (60.5%) received hybrid fixation with cemented stems. Of 38 patients, 7 (18.4%) underwent surgery under spinal anesthesia, while 31 (81.6%) had surgery under general anesthesia.

**Table 1:** Demographics of study patients.

Patient Demographics (n = 38)	Value	Standard deviation or %
Mean age (y)	91.7	±1.92
Gender		
Male	11	28.9%
Female	27	71.1%
BMI	25.2	±3.95
ASA	2.5	±0.55
ASA 1	0	0.0%
ASA 2	19	50.0%
ASA 3	18	47.4%
ASA 4	1	2.6%
Comorbidities		
HTN	32	84.2%
CAD	6	15.8%
CHF	9	23.7%
Afib	11	28.9%
DM	6	15.8%
CKD	10	26.3%
COPD	5	13.2%
Indication		
DJD/OA	35	92.1%
ONFH	2	5.3%

Afib, atrial fibrillation; CAD, coronary artery disease; CHF, congestive heart failure; CKD, chronic kidney disease; COPD, chronic obstructive pulmonary disease; DJD, degenerative joint disease; DM, diabetes mellitus; OA, osteoarthritis; ONFH, osteonecrosis of the femoral head; HTN, hypertension; SD, standard deviation.

## Results

Operative times were typically between 60 and 90 minutes with a mean operative time of 74.6 minutes ± 13.6 minutes (range: 45-106 minutes) (Table 2). There were no major perioperative complications including no incidence of myocardial infarction, pneumonia, shock, or sepsis within 7 days postoperatively. Overall, 5 patients required a blood transfusion (13.2%). Two patients experienced urinary retention. There were no surgical site infections, periprosthetic fractures, dislocations, or prosthetic joint infections within 90 days and no deaths within 30 days. Two additional patients were seen in the emergency department within 30 days, one for wound drainage and the other for a laceration, but neither required readmission.

**Table 2:** Operative outcomes following THA using the ABMS approach, n = 38.

Surgical outcomes	Value	SD or %
Received blood transfusion	5	13.2%
Operative time (min)	74.6	±13.6
Hospital length of stay (d)	2.8	±0.8
ED visit within 30 d	2	5.3%
Readmission within 90 d	2	5.3%

ABMS, anterior-based muscle-sparing approach; ED, emergency department; THA, total hip arthroplasty.

The mean length of stay (LOS) was 2.8 days ± 0.8 days (range 1.2-6.6 days). Patient disposition is outlined in Table 3. The majority of patients were discharged to a skilled nursing facility (57.9%), while 34.2% of patients returned home either with or without services. Thirty three of 33 patients for whom presurgery living situation data were available (69.7%) were discharged to a new location with a higher level of care. Only 2 patients (4.2%) were readmitted to the hospital within 90 days, one with respiratory failure unrelated to surgery and the other with a contralateral hip fracture.

**Table 3:** Patient disposition following THA, n = 38.

Patient disposition	Number	%
Home/Self-care	2	5.3%
Home with services	11	28.9%
Skilled nursing facility	22	57.9%
Acute rehab	6	15.8%

THA, total hip arthroplasty.

Patient-reported outcomes including SANE scores, HOOS JR scores, UCLA Activity scores, and PROMIS mental and physical health scores were gathered as secondary outcomes and are reported in Table 4. Response rates varied for each outcomes measure and at each follow-up time period. Mean scores were improved at 6 weeks and at 6 months to 1 year postoperatively for all outcomes measures. Compared to preoperative scores, SANE, HOOS JR, and UCLA scores were all nearly doubled at 6 weeks with marginal further improvement at 6 months to 1 year. Of note, 3 patients reported worse SANE scores and 2 patients reported worse UCLA Activity scores at 6 weeks after surgery compared to baseline, while the number of patients with SANE and UCLA scores worse than their preoperative scores dropped to 1 and 0 at 6 months to 1 year, respectively. All patients for whom HOOS JR scores were available showed increased scores postoperatively. Only PROMIS mental scores failed to show a statistically significant improvement at 6 months to 1 year postoperatively compared to preoperative scores.

**Table 4:** Patient-reported outcomes at presurgery visit, 6 weeks postsurgery, and 6 months to 1 year postsurgery.

Outcome measure	Presurgery	6 wk post surgery	Change at 6 wk	6 mo to 1 year	Change from presurgery at 6 mo to 1 y
Single Assessment Numerical Evaluation					
n	14	18	13	18	6
mean	37.4	79.9	39.1	87.1	46.7
SD	24.3	17.5	42.4	17.5	33.6
*P*-value			.02		.03
HOOS JR Interval Scores					
n	16	20	13	14	8
mean	39.3	82.2	40.9	82.3	50.395375
SD	11.4	12.8	11.4	18.3	19.438057
*P*-value			<.0001		<.0001
UCLA Activity Score					
n	18	16	13	18	10
mean	3.8	3.2	0.3	5.0	1.5
SD	1.9	0.6	3.1	1.4	1.4
*P*-value			.3		.02
PROMIS Mental					
n	17	19	12	13	9
mean	44.6	48.9	1.9	46.1	6.2
SD	6.6	6.0	3.7	6.1	16.8
*P*-value			.1		.8
PROMIS Physical					
n	17	19	12	13	8
mean	36.2	44.8	7.1	43.5	5.6
SD	3.9	6.1	5.7	6.0	1.8
*P*-value			.002		.0001

Response rates were variable and number of responding patients is listed for each score.

HOOS JR, hip dysfunction and osteoarthritis outcome score for joint replacement; PROMIS, patient-reported outcomes measurement information system.

## Discussion

Nonagenarians are a growing portion of the population and the demand for THA in this age group is growing for both elective and femoral neck fracture surgery. Our study population was similar to that of other studies looking at nonagenarians in terms of gender, medical complexity, and BMI (Table 5) [3,4,6,[8], [9], [10],^20^]. Nonagenarians tend to have multiple comorbidities, and this was true of patients in our study where 50% of patients were ASA 3 or higher. While this is reflected in several studies utilizing large databases such as those by Miric et al [4] or Sherman et al [6]; some single center studies report only a minority of patients having an ASA score of 3 or higher [6,20]. This may be due to regional differences, institutional bias regarding selection criteria for THA patients, or variation in exclusion or inclusion of patients undergoing THA for management of femoral neck fractures from study groups.

**Table 5:** Summary of contemporary literature looking at THA in nonagenarians.

Study	Antoniou 2022	Dagneaux 2021	Dimitriou 2018	Dugdale 2019	Gregory 2010	Miric 2015	Sherman 2020	Skinner 2016
Age group	90+	90-99	83-93	90+	89-96	90+	90+	90-99
N	1119	149	100	8388	31	183	1178	38
Source	ACS-NSQIP 2011-2017	Single-Center Database	Single-Center Database	NIS 2010-2014	Single-Center Database	Kaiser Permanente National TJRR 2001-2011	ACS-NSQIP 2008-2017	Single-Center Database
Mean age	Not reported	92	86 ± 2.8	90.55 ± 1.27	92	91.3 ± 1.5	Not reported	92.18
BMI	24.8 ± 4.74	26	26.5 ± 4.1	Not reported	Not reported	24.9 ± 4.0	25.9 ± 6.1	23.78
ASA scores	71.2% 3+	Mean 2; 32% 3+	Mean 2.56 50% 3+	Not reported	Median 2	66.1% 3+	71.3% 3+	Mean 2.37; 36.8% 3+
Approach	Unspecified	PA, ALA, DAA	DAA	Unspecified	Unspecified	Unspecified	Unspecified	Unspecified
Transfusion	24.3%	44%	13%	33.13%	71%	Not reported	Not reported	55.3%
LOS	4.35 d ± 5.4	4 d	5 d ± 2	6.78 d ± 4.55	12 d	3.4 d ± 1.7	4.34 d ± 6.26	8.22 d ± 4.38
Infection	0.55% (1 PJI, 5 SSI)	2% (2 PJI, 2 SSI)	2% (1 PJI, 1 SSI)	0%	3.22%	1.1% SSI	0.80%	7.89%
DVT	0.36%	1%	None reported	0.12%	6.45%	0	0.40%	0
PE	0.45%	1%	None reported	0.22%	3.22%	1	0.50%	0
UTI	3.31%	5%	None reported	5.14%	9.67%	Not reported	3.10%	Not reported
Mortality	0.98% at 30 d	6% at 90 d, 8% at 1 y	3% at 30 d, 6% at 1 y	0.19% in-hospital	6.4% at 30 d, 9.6% at 1 y	0% perioperative	0.90% at 30 d	13.2% at 1 y

Studies combining outcomes of THA and total knee arthroplasty were not included.

BMI, body mass index; LOS, length of stay; THA, total hip arthroplasty.

Our mean operative time was 74.6 minutes ( ± 13.6 minutes). This was 15 minutes less than the mean operative time published by Shah et al [21] in their retrospective review of 1313 THAs and demonstrates that the ABMS approach can be performed in a timely manner by surgeons familiar with the procedure. The normal BMIs observed in the study population may have also helped to decrease operative time relative to the general population.

We observed a transfusion rate of 13.2%. This was lower than a majority of studies (Table 5), with some authors in older studies reporting transfusion rates of up to 80% in nonagenarians [11], and other authors reporting transfusion rates around 50% in more modern studies [6,20]. Some variability between studies may be explained by the adoption of perioperative tranexamic acid, which is used routinely at our institution in all joint replacement surgeries, and may confound these findings and hinder comparison, especially to older studies. Also, while a transfusion rate of 13.2% for primary THA appears to be acceptable in this age group, it is important to note that this rate is considerably higher than the 0.5% transfusion rate observed at our institution using the ABMS approach across all age groups [18]. Nonagenarians may be at particular risk of requiring transfusion, as age, preexisting anemia, and medical complexity have all been associated with higher transfusion rates in THA [22,23]. Clinicians may also have a lower threshold to transfuse these potentially fragile patients. Though blood transfusion may be necessary in managing acute blood loss following surgery, it is not without its risks. For instance, blood transfusion following surgery for femoral neck fractures has been associated with an increased risk of infection and has been shown to be an independent risk factor for mortality at 1 year [[24], [25], [26]]. As such, efforts to avoid transfusion should be made. Institutional transfusion protocols play an important role in transfusion and may help explain our lower rates of transfusion compared to other studies. The role of surgical approach as a factor affecting transfusion rates remains unclear. In a randomized prospective study, Martin et al demonstrated a statistically significant difference in blood loss with the ABMS approach in comparison to the direct lateral approach [27]. This study is often sited when discussing decreased bleeding as a benefit of the ABMS approach and clinically reflects the theoretical lower risk of bleeding by avoiding the proximal circumflex vessels and muscle transection. A difference among approaches was also demonstrated by Yuasa et al, who, in their retrospective study of patients aged 80 years and older undergoing THA, observed a higher rate of transfusion in the posterior approach group compared to the DA approach group [28]. Dimitriou and colleagues, in their case series of outcomes of 83- to 93-year-old individuals undergoing THA utilizing the DA approach found a very similar transfusion rate to our own at 13% [3].

Literature regarding the safety of THA in nonagenarians is mixed with reported complication rates varying widely from near 0 to over 50% with medical complications alone reaching 56% in some studies [3,[9], [10], [11]]. This variation may be due to differences in follow-up time or difficulty in attributing illness with surgery in a population that tends to be more medically complex. Several recent studies have suggested increasing age is correlated with increased risk of adverse events [7,14]. This was demonstrated in a recent large database study where Antoniou et al found age greater than 90 years was associated with increased rates of major adverse events and mortality, with odds ratios of 7.6 and 3.8, respectively, when compared to patients below 90. Such findings echo previous studies by Dugdale et al [8] and Jauregui et al [13] who reported odds ratios of adverse events in nonagenarians of 1.56 and 3.39 compared to octogenarians and the general population, respectively. Similarly, Higuera et al [12] found a 40% increase in the risk of complications in THA with every additional decade of life over age 60 years. In contrast, Sherman et al [10], in a large comparative study, found no difference in rates of surgical infection, myocardial infection, stoke, pneumonia, sepsis, deep venous thrombosis, pulmonary embolism, readmission, reoperation, or mortality within 30 days of surgery in patients aged 90 years and older compared to matched patients aged 65-89 years. This is more in-line with our data which showed a very low rate of complications with no major perioperative medical or surgical complications and no deaths. Sherman et al did note a higher UTI rate of 2.8% compared to 0.9% in controls. Mortality rates also vary in the literature from 0%-2.3% perioperatively [4,29] and 6% to 13.2% at 1 year [3,6], though longer-term mortality rates appear to follow general mortality rates for this age group.

Hospital LOS is a complicated outcome and may be affected by regional differences in placement times for patients requiring a skilled nursing facility or acute rehabilitation facility, patient preferences regarding discharge disposition, and the availability of therapy services in addition to speed of patient recovery. In our study, we found an average LOS of 2.8 days ± 0.8 days which is, to our knowledge, the shortest LOS reported following THA among nonagenarians (Table 5), with 57.9% of our patients being discharged to a skilled nursing facility or acute rehabilitation facility. As stated, regional differences in rehabilitation placement likely contribute to this finding, though several studies with lower rates of nonhome discharge reported longer hospital LOS compared to our study [3,9,10]. The muscle sparing nature of the ABMS approach may have also contributed, as less muscle damage is believed to lead to faster recovery, though the time frame over which this benefit may be seen is unclear. Inaba et al [28] compared ABMS approach with the modified mini-incision direct lateral approach in a randomized prospective trial and noted lower creatine kinase levels at 1 day following surgery and better recovery of abductor muscle strength at 6 weeks in the ABMS group. Muller et al performed a similar study randomizing patients to ABMS approach or a modified direct lateral approach and found decreased gluteus medius atrophy and muscle tendon damage on magnetic resonance imaging in the ABMS group as well as lower serum myoglobin levels postoperatively [30]. These findings were reflected clinically with fewer Trendelenburg signs and higher clinical scores in the ABMS group. Similarly, Takada et al [19] studied outcomes of the DA approach to the ABMS approach in patients undergoing bilateral THA, and found significantly greater tensor fascia latae atrophy on magnetic resonance imaging and a higher incidence of lateral femoral cutaneous nerve injury on the DA side post-operatively. Herndon compared the ABMS approach to the DA approach in a retrospective study of 340 patients undergoing THA with a mean age of 61.6 and 60.4 years of age, respectively, and found no difference in LOS, distance walked with physical therapy, or disposition [31]. There is currently no study directly comparing ABMS with other approaches in this age group, though Yuasa et al [32] did note shorter LOS in patients 80 years of age and older undergoing THA via the DA approach compared to the posterior approach, supporting the theory that muscle sparing approaches may correlate with faster recovery times and shorter LOS.

Our secondary outcomes showed improvement in postoperative HOOS JR scores, UCLA Activity Scores, SANE scores, and PROMIS physical and mental health scores, supporting the efficacy of the ABMS approach to THA and efficacy of THA in nonagenarians. Multiple studies have shown significant improvements in patient-reported outcomes following THA in nonagenarians and the amount of improvement appears comparable to younger patients [3,5,6]. With respect to the ABMS approach compared to other approaches in other age groups, evidence is mixed. Martin found no difference in clinical outcomes at 1 year in comparing the ABMS approach with the direct lateral approach [27]. Takada et al [19] also found no difference in clinical outcomes at 1 year in comparing hips replaced using the DA and ABMS approaches. In contrast, D’Arringo, in a single surgeon study comparing outcomes of the ABMS, DA, and modified Hardinge approach, found superior Western Ontario and McMaster Osteoarthritis Index scores in the ABMS and DA approaches [33]. Muscle-sparing approaches may have better patient-reported outcomes early on due to faster recover as previously discussed, and this is supported in our study where the greatest increases in SANE, HOOS JR, and UCLA activity scores were seen at 6 weeks postoperatively.

### Limitations

Our study had several limitations. Lack of a control group to allow for assessment of outcomes using alternative approaches limits the ability to draw approach comparison conclusions from our study; and there is no way to control for institutional differences when comparing to previously published outcomes. Small sample size also limits our study, though our sample size is in-line with other single-center studies. Our patient-reported outcomes were particularly limited by small sample size with low response rates and variable follow-up times. The retrospective nature of our study provides a further limitation and makes our study vulnerable to selection and information bias. Modern electronic medical records allow for extensive information extraction and inclusion of patients of age 90 years and older undergoing THA for any indication and this may help mitigate these biases.

## Conclusions

Our study shows that the ABMS approach to elective THA is safe and efficacious in nonagenarians. In comparison to other studies, our group showed decreased LOS and transfusion rates as well as lower complication rates and operative times. Given its potential benefits with no clear drawbacks compared to other approaches, the ABMS approach should be considered when performing THA in elderly patients who may be at greater risk of complications and longer lengths of stay. Further research comparing the ABMS approach to other approaches, especially the DA approach which may have similar results, is warranted.

## Conflicts of interest

George Babikian, Adam Rana, and Brian McGrory are authors who are paid consultants for Smith & Nephew, Inc., and the data set for this paper came from a Smith Nephew–funded research project, data sharing agreement. The other authors declare no potential conflicts of interest.

For full disclosure statements refer to https://doi.org/10.1016/j.artd.2023.101125.

## Figures and Tables

**Conflict of Interest Statement for Sturgeon:**
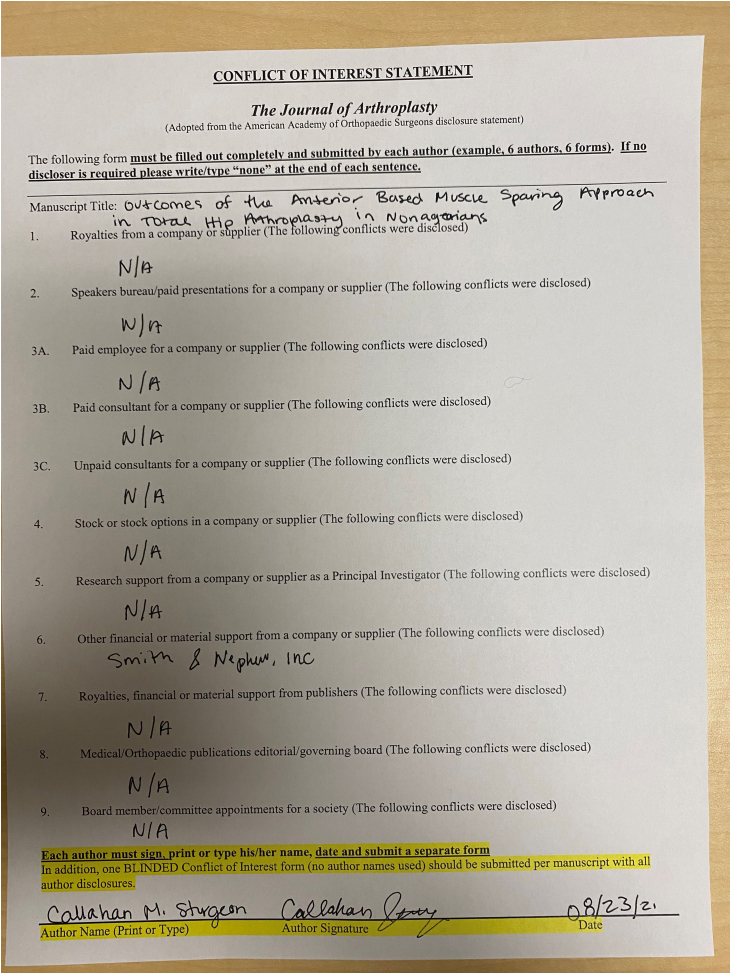


## Appendix A Supplementary Data

Conflict of Interest Statement for Rana

Conflict of Interest Statement for McGrory

Conflict of Interest Statement for Babikian

Conflict of Interest Statement for Mackenzie

Conflict of Interest Statement for Zink
