# Supplementary material for: Outcomes of the Anterior-Based Muscle-Sparing Approach in Elective Total Hip Arthroplasty in Nonagenarians
Source: Arthroplast Today. 2023 May 11;21:101125. doi: 10.1016/j.artd.2023.101125 (PMC10186483; doi:10.1016/j.artd.2023.101125)
Supplement: Conflict of Interest Statement for McGrory [file mmc2.pdf]

CONFLICT OF INTEREST STATEMENT

The Journal of Arthroplasty

(Adopted from the American Academy of Orthopaedic Surgeons disclosure statement)

The following form **must be filled out completely and submitted by each author (example, 6 authors, 6 forms)**. **If no discloser is required please write/type "none" at the end of each sentence.**

Manuscript Title: OUTCOMES OF THE ANTERIOR BASED MUSCLE SPARING APPROACH IN TOTAL HIP ARTHROPLASTY IN NONAGENARIANS

1. Royalties from a company or supplier (The following conflicts were disclosed)

   SMITH & NEPHEW, INC
   INNOMED, INC

2. Speakers bureau/paid presentations for a company or supplier (The following conflicts were disclosed)

   SMITH & NEPHEW, INC

3A. Paid employee for a company or supplier (The following conflicts were disclosed)

   NA

3B. Paid consultant for a company or supplier (The following conflicts were disclosed)

   SMITH & NEPHEW, INC

3C. Unpaid consultants for a company or supplier (The following conflicts were disclosed)

   NA

4. Stock or stock options in a company or supplier (The following conflicts were disclosed)

   NA

5. Research support from a company or supplier as a Principal Investigator (The following conflicts were disclosed)

   NA

6. Other financial or material support from a company or supplier (The following conflicts were disclosed)

   NA

7. Royalties, financial or material support from publishers (The following conflicts were disclosed)

   NA

8. Medical/Orthopaedic publications editorial/governing board (The following conflicts were disclosed)

   AAHKS, ARTHROPLASTY TODAY

9. Board member/committee appointments for a society (The following conflicts were disclosed)

   NA

Each author must sign, print or type his/her name, date and submit a separate form
In addition, one BLINDED Conflict of Interest form (no author names used) should be submitted per manuscript with all author disclosures.

BRIAN J. McGRORY, MD	[signature]	8/18/2021
Author Name (Print or Type)	Author Signature	Date
